# Assessment of 5-Hydroxymethylfurfural in Food Matrix by an Innovative Spectrophotometric Assay

**DOI:** 10.3390/ijms25158501

**Published:** 2024-08-04

**Authors:** Nadia Geirola, Simona Greco, Rosario Mare, Domenico Ricupero, Mariagiovanna Settino, Luca Tirinato, Samantha Maurotti, Tiziana Montalcini, Arturo Pujia

**Affiliations:** 1 Department of Clinical and Experimental Medicine, University “Magna Græcia” of Catanzaro, 88100 Catanzaro, Italy; n.geirola@unicz.it (N.G.); domenico.ricupero@izsmportici.it (D.R.); margiov.settino@gmail.com (M.S.); smaurotti@unicz.it (S.M.); tmontalcini@unicz.it (T.M.); 2 Department of Medical and Surgical Sciences, University “Magna Græcia” of Catanzaro, 88100 Catanzaro, Italy; simona.greco001@studenti.unicz.it (S.G.); tirinato@unicz.it (L.T.); pujia@unicz.it (A.P.); 3 Research Center for the Prevention and Treatment of Metabolic Diseases, University “Magna Græcia” of Catanzaro, 88100 Catanzaro, Italy

**Keywords:** spectrophotometry, HMF, HPLC, vinegar, Seliwanoff assay

## Abstract

Foods contaminants pose a challenge for food producers and consumers. Due to its spontaneous formation during heating and storage, hydroxymethylfurfural (HMF) is a prevalent contaminant in foods rich in carbohydrates and proteins. Colorimetric assays, such as the Seliwanoff test, offer a rapid and cost-effective method for HMF quantification but require careful optimization to ensure accuracy. We addressed potential interference in the Seliwanoff assay by systematically evaluating parameters like incubation time, temperature, and resorcinol or hydrochloric acid concentration, as well as the presence of interfering carbohydrates. Samples were analyzed using a UV–Vis spectrophotometer in scan mode, and data obtained were validated using HPLC, which also enabled quantification of unreacted HMF for assessing the protocol’s accuracy. Incubation time and hydrochloric acid percentage positively influenced the colorimetric assay, while the opposite effect was observed with the increase in resorcinol concentration. Interference from carbohydrates was eliminated by reducing the acid content in the working reagent. HPLC analyses corroborated the spectrophotometer data and confirmed the efficacy of the proposed method. The average HMF content in balsamic vinegar samples was 1.97 ± 0.94 mg/mL. Spectrophotometric approaches demonstrated to efficiently determine HMF in complex food matrices. The HMF levels detected in balsamic vinegars significantly exceeded the maximum limits established for honey. This finding underscores the urgent need for regulations that restrict contaminant levels in various food products.

## 1. Introduction

The presence of contaminants in food poses a significant challenge for both manufacturers and consumers. A wide range of factors can introduce contaminants into food products, and so gaining a deep understanding of these contaminants’ characteristics is essential for developing effective strategies to minimize their presence and ensure food safety. This knowledge empowers consumers to make informed choices and ensures the quality and safety of the food we consume daily.

Among food contaminants, hydroxymethylfurfural (HMF) has emerged as a compound of scientific interest. This carbohydrate derivative forms spontaneously during thermal processing and food preservation techniques, such as cooking or pasteurization, as a natural byproduct of the Maillard reaction [1]. For the aforementioned reasons, the HMF concentration often serves as valuable quality parameter for assessing the freshness [2], aging, or processing history of food products. As a consequence, accurate quantification of HMF provides information about quality deterioration, heat treatment intensity, and adulteration practices [3]. Data available in the literature about the effects of HMF on the human body are divergent, and the opinions are controversial. Indeed, currently there is no strong scientific evidence suggesting any direct health benefits from consuming HMF, even if some studies underlined positive effects deriving from HMF consumption [4,5]. On the other hand, there are growing concerns about the safety of consuming high concentrations of HMF in diets, and copious studies have linked excessive HMF intake to potential adverse health effects for human health, such as the possibility of causing anaphylactic symptoms by acting as agonist to histamine H1 receptor [6]. 

Driven by these scientific and health concerns, various governments have implemented regulations to limit the amount of HMF permitted in certain foods such as honey, for which a maximum allowable HMF level was established at 40 mg/kg, prioritizing consumer well-being. Consequently, numerous analytical methods have been developed to quantify HMF in honey. A recent example is the approach proposed by Besir and colleagues, who proposed the use of the Seliwanoff colorimetric assay or the various liquid chromatography methods available in literature for effectively quantifying HMF in honey [7,8]. However, despite the seemingly robust data they generate, these methods often suffer from limitations in application. In fact, they may have difficulty analyzing complex and colorful food matrices or eliminating interference caused by a high carbohydrate content, which are very frequent drawbacks in the analysis of foods such as honey.

Indeed, the Seliwanoff colorimetric assay, for instance, was originally designed precisely for estimating ketose carbohydrates such as fructose, for which HMF acts as a reaction intermediate, but it includes disaccharides like sucrose and other carbohydrates, factors leading to inaccuracies in HMF quantification. Furthermore, the focus on HMF in honey regulation overlooks the presence of significant HMF levels in other food containing high protein and carbohydrate contents, especially in long-term storage, as in case of processed and cooked meats [9,10], maple and corn syrups [11], and various fruit byproducts such as juices, concentrates, and jams [12]. Interestingly, balsamic vinegar is a condiment notoriously abundant in HMF, even in organic and traditionally produced varieties, probably due to fermentation process involved in its production [13]. However, unlike honey, balsamic vinegar remains unregulated worldwide in terms of HMF content. In addition to this, balsamic vinegar represents one of the most difficult foods to be analysed using colorimetric assays due to its intense dark color, which causes interference in the interaction with light.

Even though UV–Vis spectrophotometry and colorimetric assays are well-established techniques, they remain valuable tools for food research and analysis [14].

In this manuscript, we propose a groundbreaking, robust, and versatile method for effectively quantifying HMF in complex food matrices. The method’s parameters exhibit applicability in both spectrophotometry and high-pressure liquid chromatography (HPLC) analyses and demonstrably offer superior sensitivity and detection limits compared to previous methods documented in the scientific literature, thus allowing the characterization of various vinegars including balsamic vinegars.

## 2. Results and Discussion

With the aim of setting a selective protocol in detecting 5-hidroxymetilfurfural, all interfering parameters included in the original Seliwanoff colorimetric assay were evaluated, such as the percentage of resorcinol and hydrochloric acid in working reagent, the incubation time, and the presence of different carbohydrates in addition to the generally used fructose.

Even though resorcinol is used as excess reagent in the Seliwanoff reaction, increasing its concentration beyond 0.2% does not lead to higher absorbance values. In fact, samples with 0.1% and 0.2% resorcinol showed similar results. Interestingly, using 0.5% or 1% resorcinol actually decreased the absorbance by up to 45% (Table 1). 

In contrast to resorcinol, the concentration of hydrochloric acid and the incubation time was demonstrated to significantly affect the reaction. In detail, higher hydrochloric acid concentration and longer incubation times lead to directly proportional increases in absorbance values. In fact, using a 24% hydrochloric acid solution resulted in absorbance values five times higher compared to samples incubated with a 12% solution (Table 1). Different scientific evidence supports the data we have obtained, although generally it is preferable not to exceed 12% HCl in order to reduce the reaction exothermicity and increase safety in procedures, which is why it would be desirable to use acid doses lower than 24% *w*/*w* [7].

A similar trend was observed with the modulation of incubation time at 70 °C in the sample containing ketose carbohydrates. In detail, fructose evidenced the highest sensitivity, demonstrating a progressive rise in absorbance, while sucrose exhibited minimal reactivity for the initial 15 min of incubation, finally showing a slight increase in absorbance after 30 min of incubation. Nevertheless, the overall reactivity of sucrose remained significantly lower compared to fructose. In contrast to other carbohydrates, only negative results were obtained using glucose even up to 90 min of incubation (Table 2). 

As a function of the obtained experimental data, we decided to restrict the temperature to a maximum of 70 °C and limit the incubation time to 60 min, also optimizing the incubation time based on the hydrochloric acid concentration. This approach was adopted to prevent an excessive increase in carbohydrate sensitivity towards the assay. Our decision is corroborated by previous research from Shahidullah and Khorasani, whose findings indicated that glucose and sucrose only produced positive results in the colorimetric assay at doses 20 and 40 times higher than fructose, respectively, and required double the incubation time compared to ketosis carbohydrates [15].

Fructose solutions consistently demonstrated a response in the colorimetric assay with 12% HCl, irrespective of the sample-to-reagent ratio. However, a positive correlation was observed between the ratio and assay sensitivity for fructose detection. Notably, the 1:1 and 1:2 ratios were insufficient for detecting the minimum dose (0.3 mg/mL) of fructose, probably because the minor working reagent leads to a smaller quantity of available acid. Conversely, samples prepared with 1:4 and 1:8 ratios yielded quantifiable results. Interestingly, the 1:8 dilution resulted in absorbance values approximately twofold higher than those obtained with the 1:4 dilution, despite the greater sample dilution. However, the 1:8 samples exhibited poor repeatability, evident from the high standard deviation values that sometimes approached the mean absorbance value. In contrast, the 1:4 ratio produced more reproducible and reliable data, as evidenced by the significantly lower standard deviation values (Table 3). Therefore, it seemed appropriate to choose a ratio of 1:4 for subsequent experiments, with the aim of preserving a sufficient sensitivity to the assay and reducing wastes of working solution. Our proposal is also supported by data previously described in the literature because several research groups efficiently used a ratio of 1:4 as the best compromise for the execution of the traditional assay [7].

Standard solutions of 5-hydroxymethylfurfural (HMF) presented a main peak in the near-UV region, specifically around λ~285 nm, a parameter that allowed quantification both by spectrophotometric analyses and by HPLC. Additionally, HMF reacts with resorcinol in the presence of an acidic catalyst, forming a colored complex detectable and quantifiable by UV–Vis spectrophotometry or HPLC at wavelengths exceeding 450 nm, albeit with varying sensitivity. Notably, unlike carbohydrates, HMF efficiently binds to resorcinol even at hydrochloric acid concentrations lower than 12%; indeed, it was observed to react with resorcinol even at 1% HCl. However, positive results at this concentration were only obtained with high HMF doses, while lower concentrations were quantifiable exclusively by HPLC due to its superior sensitivity. The observed phenomenon suggested that an alkaline food matrix might interfere with the assay kinetic due to the increase in pH value, which can neutralize HCl, thus precluding HMF to complex resorcinol.

**Figure 1:**
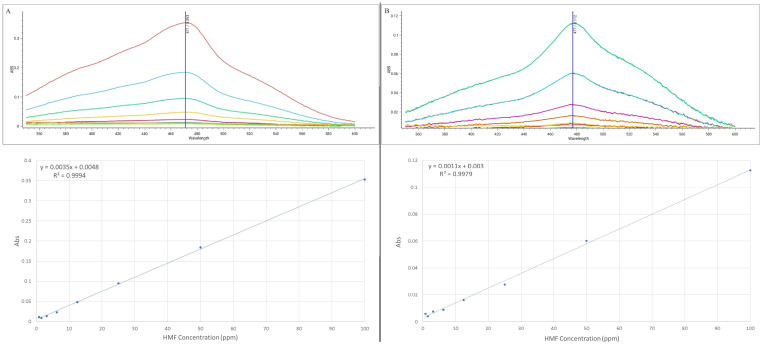
Spectrophotometric scanning results of HMF (concentration range 100 ppm–0.078 ppm) incubated at 70 °C with a mixture containing 3% HCl and 0.1% resorcinol. The samples were incubated 30 (panel (**B**)) minutes or 60 min (panel (**A**)) before the analyses.

Positive dose-dependent results were obtained using HMF and 3% HCl solutions, a setting that allowed the quantification of the furfural both by spectrophotometry and HPLC in samples ranging between 100 ppm and 6.25 ppm. Interestingly, the reduction in HCl percentage involved in a shift of the main peak of HMF complex between 471 nm < λ < 478 nm and a reduction in the limit of quantification, with the 3.125 ppm sample insensitive to the protocol proposed after 30 min of incubation (Figure 1). Nevertheless, the possibility of modulating the incubation time made it possible to increase the HMF sensitivity to the assay and linearity of data obtained, with samples incubated for 60 min showing almost three-fold higher absorbance values and 3.125 ppm samples becoming clearly detectable and quantifiable in comparison with 30 min incubation samples (Figure 1).

In detail, in spectrophotometric analyses, all samples showed linearity in absorbance results with a concentration between 100 ppm and 6.25 ppm after 30 min of incubation, while samples maintained at 70 °C up to 60 min exhibited increased sensitivity and preserved linearity up to the lower limit of 3.12 ppm (Figure 1).

**Figure 2:**
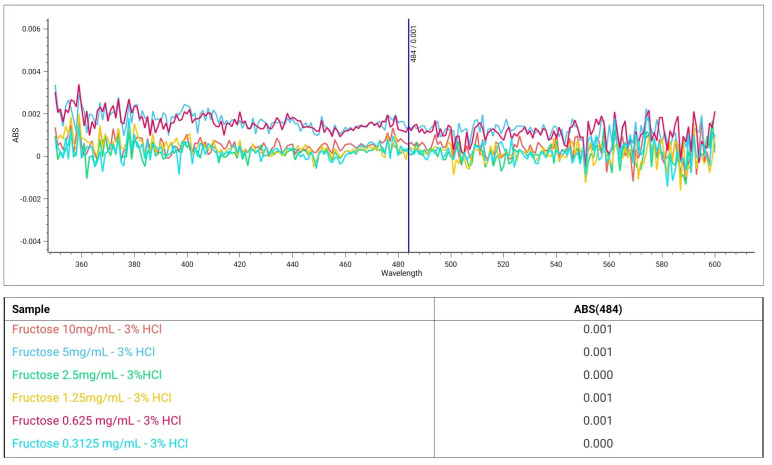
Spectrophotometric analyses of fructose (0.3–10 mg/mL) after the execution of the modified Seliwanoff assay. All samples showed negative results for the protocol.

Conversely, dilute acid solutions failed to react with carbohydrates, even with the most reactive fructose, which evidenced negative results after incubation with resorcinol solutions containing 3% HCl concentrations, regardless of the sugar doses (Figure 2). The negative results obtained with fructose support the reliability of the proposed protocol and validate the absence of possible interference during the analysis of food matrices rich in carbohydrates.

**Figure 3:**
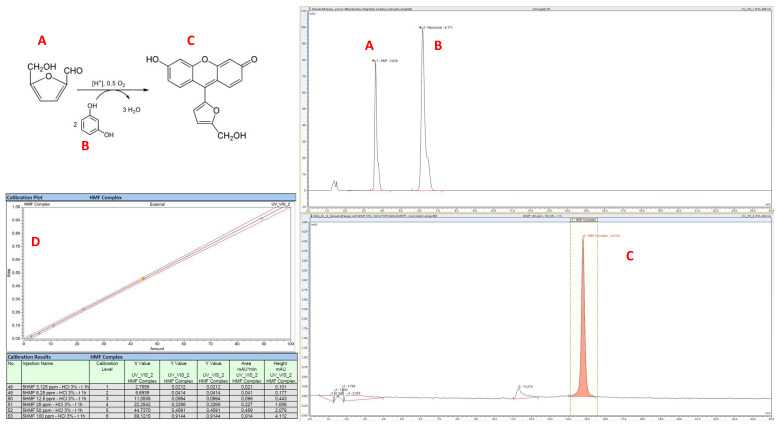
HPLC analyses of HMF (**A**) and resorcinol (**B**) before and after performing the colorimetric assay, with newly derived characteristic complexes (**C**). Calibration curve obtained at HMF concentration between 3.125 ppm and 100 ppm (panel (**D**)—r^2^ > 0.99).

HPLC analyses were used to prove the effectiveness, precision, and accuracy of the results obtained by UV–Vis spectrophotometry. In detail, standard hydroxymethylfurfural and resorcinol were efficiently separated and detected at 285 nm, showing a retention time (RT) of approximately 3.2 ± 0.4 and 5.4 ± 0.5 min, respectively. The conjugation products between HMF and resorcinol were detectable and quantifiable in HPLC analyses at 484 nm, with the onset of one or more peaks with an RT between 11 and 16 min, which were also weakly detectable at 285 nm in high-dose samples (Figure 3).

Interestingly, the analyses of the colored complex at 484 nm sometimes evidenced the presence of a main peak with a ~15 min retention time and a second and proportional peak with an RT of ~11 min, a circumstance more frequent in fructose samples and using high HCl doses, while cases in which the peak had an RT of ~11 min showed higher intensity and area values.

The observed phenomenon can be explained through the formation of different conjugation derivatives between HMF and resorcinol. This hypothesis is supported by data previously published by Sánchez-Viesca and Gómez, who explained the reactivities of the Seliwanoff test and demonstrated that at least three different structures are involved and responsible for the onset of the red color in solutions [16].

**Figure 4:**
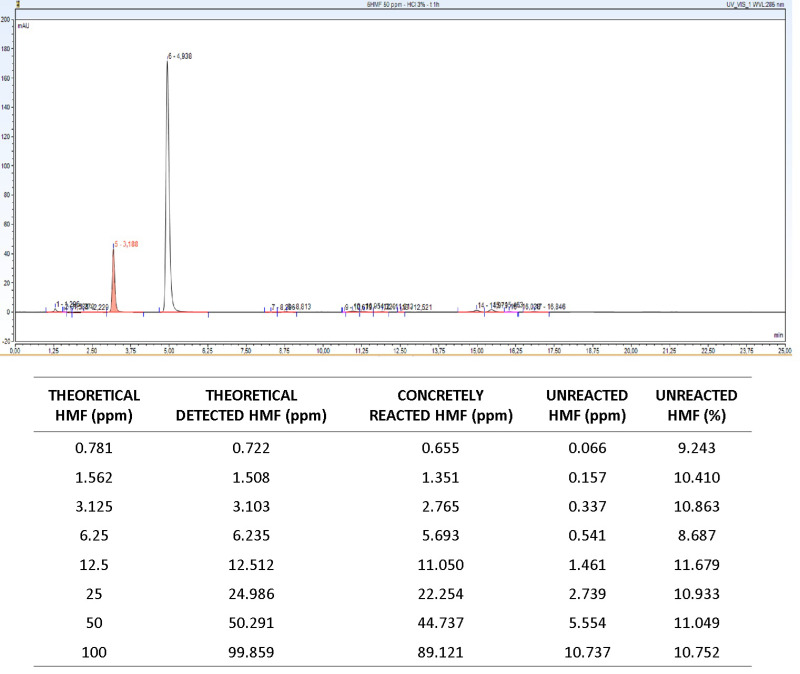
HPLC quantification of unreacted HMF in samples with concentration between 100 ppm and 0.781 ppm.

As in all chemical reactions, a part of the HMF contained in the samples may not conjugate to resorcinol. Altering the reaction parameters could potentially lead to increased levels of unreacted HMF, consequently compromising the efficiency of the established method. HPLC analyses corroborated the data obtained via UV–Vis spectrophotometry and facilitated the quantification of the non-conjugated HMF fraction. Notably, regardless of the concentration analyzed, roughly 90% of the incubated HMF effectively formed a complex with resorcinol, resulting in a colored solution quantifiable by both HPLC and UV–Vis spectrophotometry (Figure 4).

The amount of unreacted HMF in the proposed protocol almost overlapped with that of the doses detected with the conventional Seliwanoff method. In detail, regardless of the parameters adopted, ~10% of molecules took no part in the Seliwanoff condensation reaction, even when using ketose carbohydrates, in fructose samples (Supplemental Figure S1).

The developed spectrophotometric protocol was used to quantify the HMF contained in different balsamic vinegars widely consumed in the Italian peninsula, condiments representing some of the most difficult food matrices to be analyzed due to their intense dark black color. The rationale for choosing balsamic vinegar is specifically its distinct and intensely dark color, which poses challenges for analysis using colorimetric methods. In addition, the acidic pH of balsamic vinegar offers an advantage in this approach, as lower pH values are demonstrated to enhance the assay’s sensitivity. Therefore, acidic or neutral foods are preferable for the proposed protocol. Additionally, the acidity in balsamic vinegar mainly derives from acetic acid and succinic acid [17]. These organic acids have acid dissociation constants of 1.74 × 10^−5^ and 6.21 × 10^−5^, respectively. As a result, their contribution to the release of H^+^ ions in aqueous solutions is minimal compared to the acidity from HCl contained in the assay working reagent.

**Figure 5:**
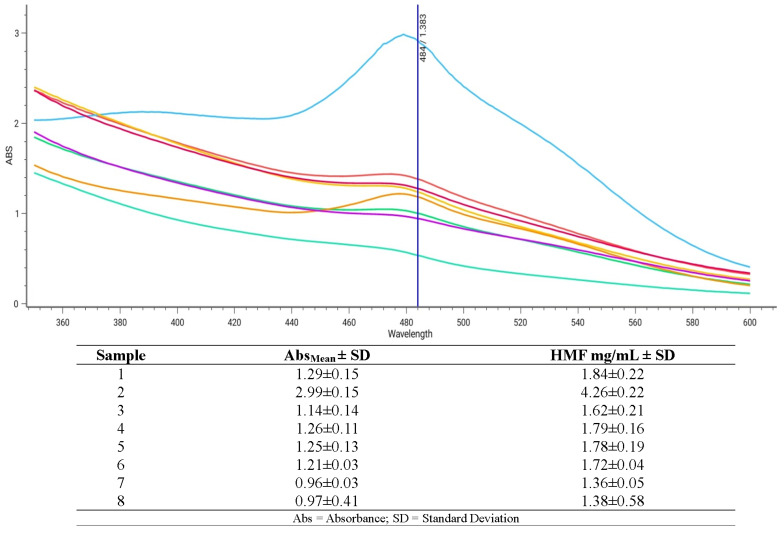
Spectrophotometric quantification of HMF contained in vinegar samples.

The colorimetric assay gave positive results with all balsamic vinegar samples. In detail, samples were shown to contain a mean HMF value of 1.97 ± 0.94 mg/mL, with the best sample containing only 1.36 ± 0.05 mg/mL of HMF, while the worst sample had 4.26 ± 0.22 mg/mL (Figure 5).

Interestingly, the sample with the highest amount of HMF, according to what was reported on the label, was organic and produced with traditional methods, factors which are not synonymous with excellent quality in industrial and commercial products. In addition to production methods, other aspects and circumstances can influence the quantity of contaminants in foods, including transport methods and storage conditions. In addition to this, almost half of the samples were also organic, and one of them was the balsamic vinegar with the lowest amount of HMF.

Finally, with the aim of demonstrating the method’s applicability to various food matrices, the outlined protocol was employed to measure HMF levels in different honey samples, which are frequently analyzed for furfural content. The results obtained confirmed the feasibility of this novel approach in analyzing various foods, including those rich in carbohydrates (Supplemental Figure S2).

## 3. Materials and Methods

### 3.1. Materials

Hydroxymethylfurfural (HMF), fructose, resorcinol, hydrochloric acid (HCl), formic acid, methanol (MeOH), and acetonitrile (ACN) were purchased from Sigma-Aldrich (Sigma-Aldrich—Merck KGaA, Darmstadt, Germany). All other solvents and reagents were of analytical grade (Cartlo Erba, Milan, Italy).

### 3.2. Vinegar Sample Preparation

Balsamic vinegars for tests were produced by different companies and randomly bought in local markets. All samples were centrifuged (Eppendorf Centrifuge 5500 – Eppendorf, Milan, Italy) at 3000 rpm for 10 min and finally filtered through 0.22 µm filters before being analyzed. Additionally, all samples were appropriately diluted with milliQ^®^ water before the analyses.

### 3.3. HMF Quantification

All detection and quantification results for hydroxymethylfurfural (HMF) and its derivatives were obtained and compared by two different approaches based on a modified version of the Seliwanoff assay previously described in the literature [7]. All samples were analyzed both by UV–Vis spectrophotometry and high-performance liquid chromatography (HPLC) as better described below.

### 3.4. Spectrophotometric Quantification 

The UV–Vis spectrophotometer apparatus is a ThermoScientific—Genesys^®^ 150 (ThermoScientific, Milan, Italy) equipped with Exacta Optec quartz cuvettes. Suitable calibration curves were used for quantitative analyses in all cases and obtained using appropriate standard solutions (r^2^ ≥ 0.98 in all cases). Analyses were conducted in triplicate and appropriate dilutions applied when necessary in order to not exceed the limit of the absorbance value according to the Lambert–Beer law. A blank sample for each protocol was applied. When possible, scanning in a wide wavelength range (190 < λ< 700 nm) was performed to detect main peaks. The sensitivity was set to a medium value, with absorbance detection every 1 nm.

For the colorimetric assay, a proper working reagent was prepared by dissolving resorcinol in HCl aqueous solution at different concentrations. Subsequently, samples and working reagent were mixed and incubated with the temperature set to 70 °C. Finally, samples obtained were incubated in ice with the aim of stopping the reaction and the absorbance measured at λ ~ 484 nm.

### 3.5. HPLC Analyses

The HPLC apparatus consisted of a ThermoFisher Scientific Vanquish System Base furnished with a quaternary pump, a split sampler, a thermoregulated column compartment, and a UV–Vis detector (ThermoFisher Scientific—Rosano, MI, Italy). The reverse-phase C18 column was an Acclaim^®^120 with 100 mm length and 5 µm silica particle size, with the temperature set to room temperature. 

The mobile phase was formic acid aqueous solution (0.1%) and acetonitrile (ACN). The organic phase increased from 10% up to 30% in 10 min and then preserved this ratio until the end of the analysis. UV–Vis signals were acquired using double-wavelength measurement at 285 nm and 484 nm. The quantification and recognition of the peaks occurred through comparison with the signals generated by the standard molecules. Each calibration line was obtained with a concentration decrease of at least 5 points, and when possible 8 points were used (r^2^ > 0.98). Samples of 5 µL were injected into the HPLC apparatus with the total acquisition time set to 25 min. All samples were filtered through 0.22 µm cellulose filters before being injected into the HPLC.

### 3.6. Method Validation Parameters

In the validation study of the proposed spectrophotometric method, the linearity, selectivity, limit of detection (LOD), and limit of quantification (LOQ) were evaluated.

The specificity of the method was determined by running blank samples of milliQ^®^ water and 0.1% resorcinol solutions with different quantities of hydrochloric acid during all analyses. Blank runs confirmed the absence of interfering peaks at the retention time of HMF or its complex.

The peak area of each concentration was calculated. Concentrations ranged between 100 and 0.7 ppm with HMF standard solutions and between 100 and 3.125 ppm in the colorimetric assay. The calculated coefficients of determination (r^2^), obtained when plotting the peak area against concentration, were 0.99976 and 0.99999 respectively, thus suggesting good linearity between the peak area and concentration in the aforementioned concentration range. The equations of the regression line derived from the concentration standards were y = 0.7383*x* + 0.0272 and y = 0.0104*x* − 0.0112, respectively, obtained with 8 standard HMF solutions and 6 samples from the colorimetric assay.

The limit of detection (LOD) and limit of quantification (LOQ) were calculated using the following equations:

LOD = 3 Sa/m.

LOQ = 10 Sa/m

where Sa = standard deviation of the intercept of the regression line and m = slope of the calibration curve. The limit of detection was 2.225 ppm, while the limit of quantification had a value of 7.417 ppm.

These limits are similar to those previously published in the literature for spectrophotometric analyses but significantly lower than those previously used in chromatography analyses [18] for the efficacious quantification of HMF in vinegars.

## 4. Conclusions

This study highlights the importance of quantifying food contaminants, like HMF, with the aim of ensuring consumer health. The newly developed UV–Vis spectrophotometry method offers a rapid, cost-effective tool for accurate food analysis using colorimetric assays. While careful optimization is crucial to avoid interference in colorimetric assays, complexation with resorcinol in a weakly acidic environment proved to be a successful approach for determining HMF in complex food matrices, such as balsamic vinegars. Our findings reveal significant HMF levels in balsamic vinegars, regardless of origin or production method. This emphasizes the need for regulations limiting HMF content in various foods, similar to the existing laws for honey.

## Figures and Tables

**Table 1:** Influence of resorcinol and HCl percentage in standard Seliwanoff assay.

Sample	Amount mg/mL	Time (min.)	HCl	Resorcinol	W.S. Ratio	Abs_Mean_ ± SD
Fructose	0.3125	30	12%	0.1%	1:4	0.0037 ± 0.0001
				0.2%		0.0038 ± 0.004
				0.5%		0.0026 ± 0.008
				1%		0.0021 ± 0.001
	0.3125	30	12%	0.1%	1:4	0.013 ± 0.009
	0.625					0.023 ± 0.009
	1.25					0.048 ± 0.014
	2.5					0.097 ± 0.035
	5					0.205 ± 0.067
	0.3125		24%			0.079 ± 0.047
	0.625					0.131 ± 0.082
	1.25					0.279 ± 0.176
	2.5					0.599 ± 0.419
	5					1.474 ± 1.109

W.S. = working solution; Abs = absorbance; SD = standard deviation.

**Table 2:** Influence of incubation time on different carbohydrates.

Time	Fructose * Abs_Mean_ ± SD	Sucrose * Abs_Mean_ ± SD	Glucose * Abs_Mean_ ± SD
15′	0.0034 ± 0.0002	−0.004 ± 0.010	0.006 ± 0.002
30′	0.0221 ± 0.0044	0.009 ± 0.006	−0.018 ± 0.006
45′	0.055 ± 0.0050	0.034 ± 0.003	0.004 ± 0.001
60′	0.0918 ± 0.0085	0.053 ± 0.001	−0.019 ± 0.012
75′	0.1225 ± 0.0056	0.077 ± 0.003	−0.008 ± 0.0010
90′	0.1531 ± 0.0142	0.093 ± 0.005	−0.019 ± 0.006

Abs = absorbance; SD = standard deviation; * = concentration 0.3 mg/mL.

**Table 3:** Influence of working solution ratio in the assay’s sensitivity.

W.S. Ratio	Fructose (mg/mL)	Abs_Mean_ ± SD	W.S. Ratio	Fructose (mg/mL)	Abs_Mean_ ± SD
1:1	0.3	n.a.	1:4	0.3	0.013 ± 0.009
	0.6	0.012 ± 0.001		0.6	0.023 ± 0.009
	1.2	0.034 ± 0.015		1.2	0.049 ± 0.014
	2.5	0.049 ± 0.004		2.5	0.097 ± 0.035
	5	0.118 ± 0.02		5	0.205 ± 0.067
1:2	0.3	n.a.	1:8	0.3	0.029 ± 0.030
	0.6	0.016 ± 0.001		0.6	0.061 ± 0.062
	1.2	0.043 ± 0.019		1.2	0.189 ± 0.122
	2.5	0.065 ± 0.004		2.5	0.220 ± 0.229
	5	0.135 ± 0.029		5	0.493 ± 0.518

W.S. = working solution; Abs = absorbance; SD = standard deviation.

## Data Availability

The original contributions presented in the study are included in the article/Supplementary Material, further inquiries can be directed to the corresponding author.

## Supplementary Materials

The following supporting information can be downloaded at: https://www.mdpi.com/article/10.3390/ijms25158501/s1.
