# Supplementary figures and images for: Assessment of 5-Hydroxymethylfurfural in Food Matrix by an Innovative Spectrophotometric Assay
Source: Int J Mol Sci. 2024 Aug 4;25(15):8501. doi: 10.3390/ijms25158501 (PMC11313681; doi:10.3390/ijms25158501)

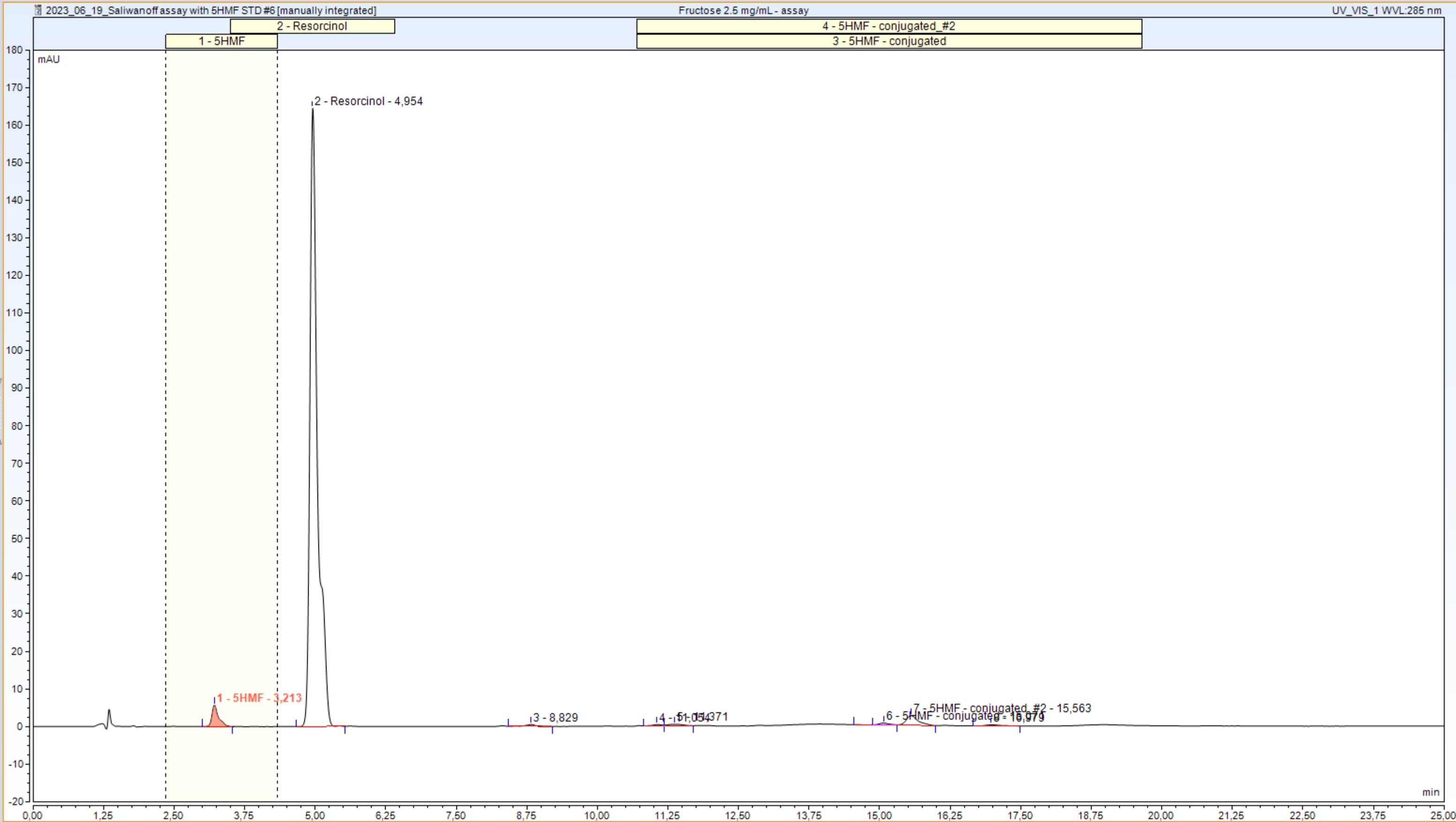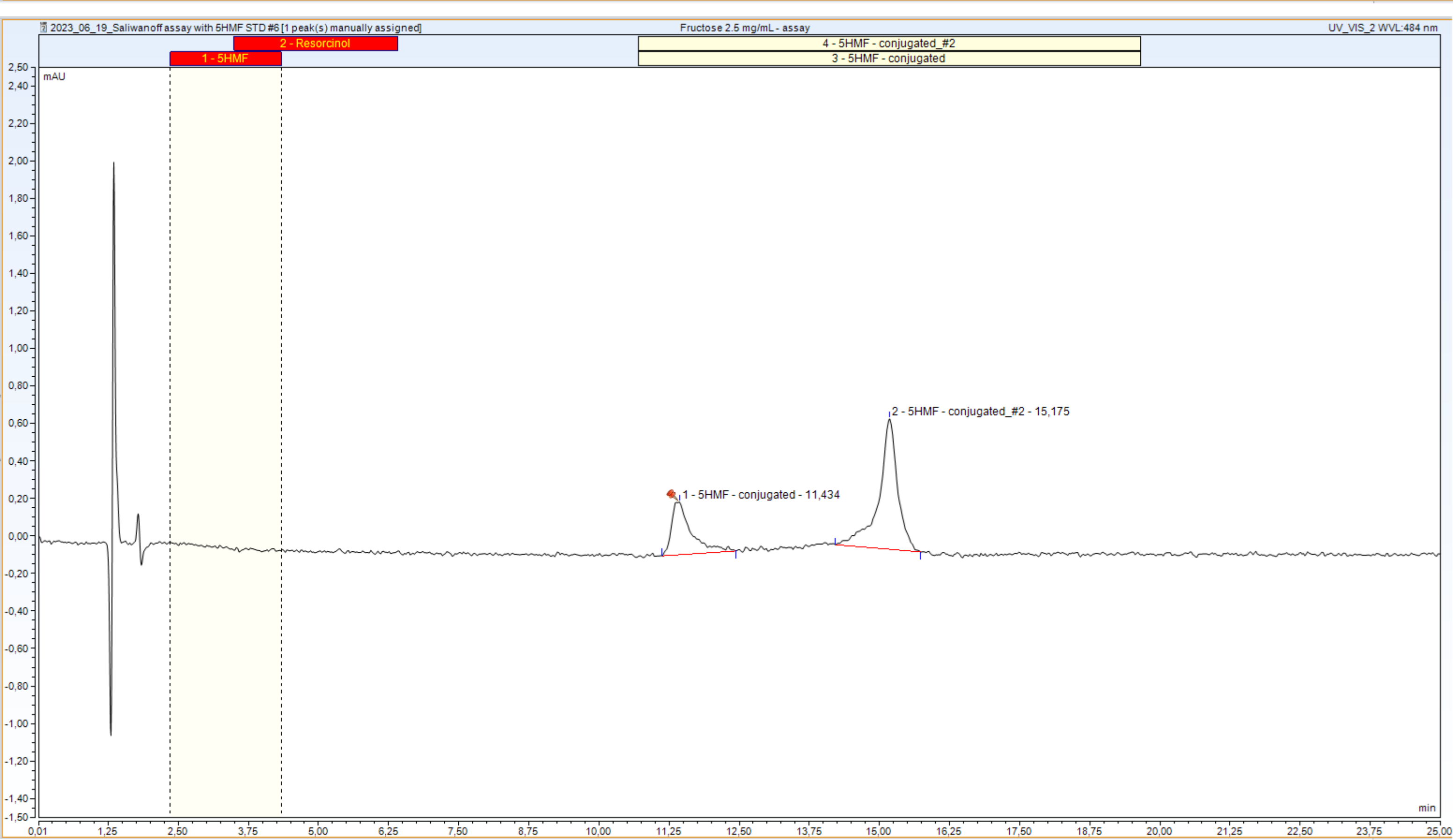

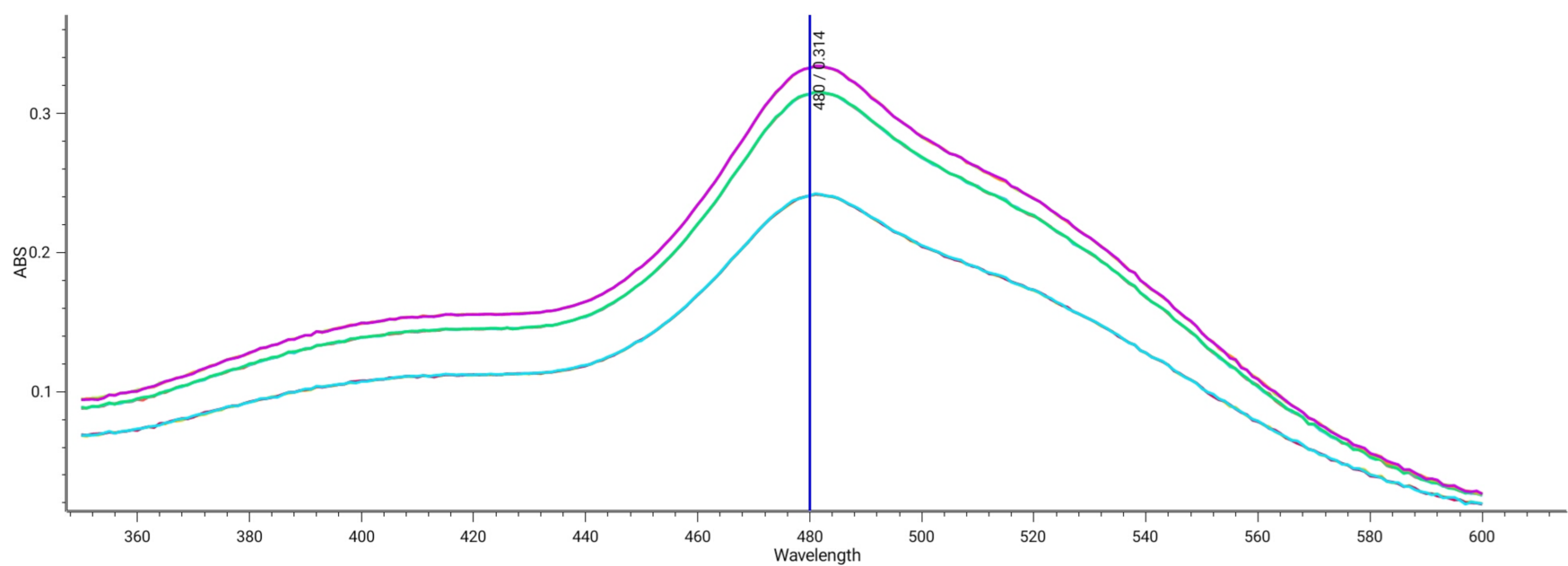

| Wavelength (nm) | Abs 484.0 | Abs <sub>MEAN</sub> ± SD | mg/mL ± SD      |
|-----------------|-----------|--------------------------|-----------------|
| Honey #1        | 0.31343   | 0.31351 ± 0.00011        | 6.4176 ± 0.0021 |
|                 | 0.31364   |                          |                 |
|                 | 0.31347   |                          |                 |
| Honey #2        | 0.23987   | 0.24015 ± 0.00026        | 5.0068 ± 0.0049 |
|                 | 0.24021   |                          |                 |
|                 | 0.24038   |                          |                 |
| Honey #3        | 0.33189   | 0.33197 ± 0.000075       | 6.7725 ± 0.0014 |
|                 | 0.33204   |                          |                 |
|                 | 0.33198   |                          |                 |

Supplement: Supplementary file 1 [file ijms-25-08501-s001.zip › ijms-3103125-supplementary.pdf]
